# Nutrition, Nutritional Status and Functionality

**DOI:** 10.3390/nu15081944

**Published:** 2023-04-18

**Authors:** Diego Fernández-Lázaro, Jesús Seco-Calvo

**Affiliations:** 1 Department of Cellular Biology, Genetics, Histology and Pharmacology, Faculty of Health Sciences, University of Valladolid, Campus of Soria, 42004 Soria, Spain; 2 Neurobiology Research Group, Faculty of Medicine, University of Valladolid, 47005 Valladolid, Spain; 3 Institute of Biomedicine (IBIOMED), Universidad de León, 24071 León, Spain; 4 Department of Physiology, University of the Basque Country, 48940 Leioa, Spain

A good quality of life means obtaining adequate nutrition and regular physical activity. This combination also reduces the risk of developing many chronic diseases while increasing one’s level of physical performance. These aspects are critical for the population. Therefore, within the field of physical activity, we must focus on the influence of nutrition on the health, performance, and recovery of a subject. Nutrition and regular physical activity are especially key for the health of the elderly, people with comorbidities, and athletes. Adequate nutritional intervention employs elements of nutritional assessment and ergo-nutritional supplementation in addition to daily diets. The nutritional status of an individual can be defined as the result between the nutritional intake received and the nutritional demands, and should allow for the utilization of nutrients to maintain reserves and compensate for losses. Given the diversity of factors and the variability of mechanisms involved in the nutritional balance of every person, it is necessary to resort to measures that guide us with respect to our nutritional status. If we are rigorous, the more techniques we use, the more accurate our final assessment will be. However, it is important to set the objective of this nutritional assessment so as not to waste time and money on unnecessary analyses.

The concept of the evaluation of nutritional status has a very wide range of characteristics and applications. From the point of view of medicine, it is essential to know the nutritional status of the largest percentage of its population as possible, especially regarding vulnerable groups such as the elderly and different groups with specific diseases, to obtain a prognosis and to be able to intervene in the course of the disease to avoid complications. The high physical demands of athletes who want to improve their performance mean that regular nutrition is insufficient. Therefore, it is necessary to use ergo-nutritional aids that cover the nutritional requirements of athletes and optimize their health and sports performance, which are also measurable with rational nutrition assessment tools. Dietary supplements are substances that add nutrients to the diet with potential nutritional, biological, and physiological effects. In 2022, the world trade in dietary supplements was valued at USD 150 million, and the forecast for the next decade estimates an annual growth rate of 8.6%, meaning that exponential growth is expected in the next five years. 

This Special Issue of *Nutrients*, entitled “Nutrition, Nutritional Status and Functionality”, has gathered together five manuscripts [1,2,3,4,5]. Two manuscripts (40.0%) analyzed the nutritional status of Italian and Spanish population cohorts [1,5]. These studies used nutritional status assessment tools such as the modified World Cancer Research Fund/American Institute for Cancer Research (mWCRF/AICR) scoring system [1] and the Mini Nutritional Assessment (MNA) [5]. Mirizzi et al. [1] studied the association of lifestyle, according to mWCRF/AICR, with all causes related to diseases of the digestive system (related to DDS) and cardiovascular diseases, as well as those related to mortality, including CVD-related, cancer-related, and other cause-related mortality. Starting in 2005, they used a cohort of 5271 participants (≥18 years) who were followed up with until 2020. In southern Italy, where the Mediterranean diet is the usual way of eating, there was high adherence to the mWCRF/AICR score. This meant that adherence to the Mediterranean diet had a remarkable protective effect on all-cause mortality in both the male and female sub-cohorts. These researchers applied the mWCRF/AICR tool in an innovative way in the Mediterranean population, obtaining valid and precise estimates. In addition, these findings could consolidate mWCRF/AICR as a clinical assessment system in both qualitative and quantitative domains of diet, as well as for overall lifestyle, and could be used to compare lifestyle habits in different populations to enhance nutritional interventions [1]. Mugica-Errazquin et al. [5] conducted a cohort study on 105 elderly people from 13 nursing homes in Spain. These investigators [5] evaluated potential associations between nutritional status, as measured by the Mini Nutritional Assessment (MNA), and parameters of functional status, physical performance, physical activity, and frailty, as well as comorbidity and body composition. Mugica-Errazquin et al. [5] obtained some results with the MNA questionnaire that were positively correlated with the Barthel index, hand grip strength, physical performance, and absolute muscle power, and were negatively correlated with dynamic balance and fragility. Therefore, this study highlighted the importance of having an optimal nutritional status that significantly influences the set of biomarkers that decisively influence a better quality of life for older adults living in nursing homes.

The three remaining studies (60.0%) in this Special Issue investigated the use of supplements in athletes; Fernández-Lázaro et al. [4] studied professional cyclists and Wirnitzer et al. [2,3] studied endurance runners. Fernández-Lázaro et al. [4] demonstrated that their multi-ingredient supplements (MIPSs), which were formulated and manufactured by these researchers, increase the effects at the physiological level and the integrated metabolic response in exercise when ingested before or after training. Thirty elite male cyclists participated in a ten-week randomized non-placebo-controlled trial that tested supplementation with their MIPS. It was found that the administration of the MIPS either pre- or post-training significantly influenced the attenuation of muscle damage and the regulation of anabolic/catabolic hormonal behavior. These functions cause a better state of health for all cyclists, and therefore, a better performance. These results were substantially greater when the MIPS was administered post-training [4]. The two studies by Wirnitzer et al. [2,3] were a part of the results of the NURMI study (Step 2), which investigated and compared the intake of supplements between female and male distance runners (10 km, half marathon, and (ultra) marathon) and the potential associations with the type of diet and the race distance. In the first study [3], whose sample was 220 endurance runners (127 women and 93 men), the most important results were that 54.3% of female runners and 47.3% of male runners reported taking a supplement regularly. Therefore, gender cannot be considered a strong modulator of supplement intake among different groups of endurance runners [3]. The second study by Wirnitzer et al. [2] evaluated supplement intake among vegan, vegetarian, and omnivorous distance runners and its relationship with age, gender, and race distance. These investigators have described a higher prevalence of vitamin supplement use in vegan (66%) endurance runners compared to vegetarians (25%) and omnivores (30%) [2].

The diversity of articles published in this Special Issue [1,2,3,4,5] highlights the use of nutritional status assessment tools in populations following the Mediterranean diet and their relationship with health status and/or absence of disease, as well as the relevance of functional status in relation to nutritional status in older adults. In addition, two studies reported that supplement consumption is similar among different groups of athletes and that the use of MIPSs improves health status and sports performance in professional cyclists.
